# Skewed X inactivation is associated with phenotype in a female with adrenal hypoplasia congenita

**DOI:** 10.1136/jmg.2007.055129

**Published:** 2008-08-26

**Authors:** M G Shaikh, L Boyes, H Kingston, R Collins, G T N Besley, B Padmakumar, O Ismayl, I Hughes, C M Hall, C Hellerud, J C Achermann, P E Clayton

**Affiliations:** 1 Department of Endocrinology, Royal Manchester Children’s Hospital, Manchester, UK; 2 Regional Genetic Service, St Mary’s Hospital, Manchester, UK; 3 Willink Biochemical Genetics Unit, Royal Manchester Children’s Hospital, Manchester, UK; 4 Department of Paediatrics, Royal Oldham Hospital, Oldham, UK; 5 Department of Neurology, Royal Manchester Children’s Hospital, Manchester, UK; 6 The Sahlgrenska Academy, Dept of Clinical Chemistry and Transfusion Medicine, Goteborg University, Sweden; 7 Developmental Endocrinology Research Group, UCL Institute of Child Health, London, UK

## Abstract

Adrenal hypoplasia congenita (AHC) can occur due to deletions or mutations in the *DAX 1* (*NR0B1*) gene on the X chromosome (OMIM 300200). This form of AHC is therefore predominantly seen in boys. Deletion of the *DAX 1* gene can also be part of a larger contiguous deletion including the centromeric dystrophin and glycerol kinase (GK) genes. We report a girl with a de novo deletion at Xp21.2 on the maternal chromosome, including *DAX1*, the GK gene and 3′ end of the dystrophin gene, who presented with salt losing adrenal insufficiency and moderate developmental delay, but relatively mild features of muscular dystrophy. Investigation using the androgen receptor as a marker gene identified skewed inactivation of the X chromosome. In the patient’s leucocytes, the paternal X chromosome was completely inactive, but in muscle 20% of the active chromosomes were of paternal origin. Thus skewed X inactivation (deletion on the active maternal X chromosome with an inactive paternal X chromosome) is associated with AHC in a female. Variability in X inactivation between tissues may account for the pronounced salt loss and adrenal insufficiency but mild muscular dystrophy.

Adrenal hypoplasia congenita (AHC) is a disorder of adrenal development resulting in primary adrenal failure in early infancy or childhood. This condition is inherited in two forms, either through autosomal recessive inheritance—for example, due to mutations in steriodogenic factor-1 (*NR5A1*)—or more commonly through X-linked inheritance.^1^^–^4 The X-linked form of AHC is due to a deletion or mutation in the gene encoding *DAX1* (*NR0B1*) and is associated with hypogonadotropic hypogonadism.^4^ X-linked AHC may occur in isolation or as part of a contiguous gene deletion syndrome at Xp21 including genes for Duchenne muscular dystrophy (DMD) and glycerol kinase deficiency (GKD). Affected boys present with adrenal failure, early muscle weakness, glyceroluria and delayed development.^5^^–^7 Occasionally loss of the telomeric locus for IL1RAPL1 is also associated with developmental delay.^8^

Rare cases of delayed puberty have been reported in female carriers of *DAX1* mutations, which are postulated to be the result of non-random inactivation of one X chromosome,^1^ but detailed molecular investigations have not been reported and these women did not have any adrenal features. We report the first case of a girl with salt losing AHC, mild dystrophinopathy and GKD associated with confirmed skewing of X inactivation of variable degree in different tissues.

## SUBJECTS AND METHODS

### Patient

A female child of unrelated white European parents presented at 8 days of age with lethargy and pyrexia, and was found to be hyponatraemic (125 mmol/l) and hyperkalaemic (9.4 mmol/l). A random cortisol was inappropriately low at 238 nmol/l and a synacthen test confirmed a low basal cortisol value (105 nmol/l) rising to an unsatisfactory peak of 420 nmol/l. The renin concentration was elevated at >77 nmol/h/l (normal range 0.38–2 nmol/h/l (supine)), while the aldosterone value was low at 80 pmol/l (normal range 1000–6000 pmol/l). A subsequent adrenocorticotrophic hormone (ACTH) concentration was also high at >220 pmol/l (normal range 0–10 pmol/l). She was commenced on hydrocortisone, fludrocortisone and sodium supplements. Her karyotype was confirmed as 46XX, and a pelvic ultrasound demonstrated the presence of normal internal female reproductive organs. Only one adrenal gland was identified, probably as the other gland was very poorly developed. Mutations in the genes encoding steriodogenic acute regulatory protein (StAR) and steriodogenic factor 1 (SF-1) were excluded.

From the age of 1 year there were concerns about her development as she was failing to reach her motor milestones and speech was delayed. At the age of 20 months she was still unable to stand and generalised hypotonia was present with notably reduced reflexes. Creatinine kinase was found to be very high at 8721 IU/L (normal value 25–180 IU/L). A muscle biopsy demonstrated a significant abnormality in her dystrophin distribution, consistent with being a manifesting carrier of muscular dystrophy. Dystrophin 1 was present in most fibres, but dystrophins 2 and 3 were only present in occasional fibres. In addition, increased concentrations of glycerol were found on urine organic acid analysis.

Her muscular dystrophy remained mild: at the age of 7 years she is able to walk a reasonable distance without difficulty, although she does occasionally complain of pain in her calf muscles.

She is currently on hydrocortisone (10 mg/m^2^/day) and a relatively large dose of fludrocortisone (250 μg/m^2^/day) on which she remains well, maintaining normal electrolytes and renin concentrations.

### Molecular investigations

Fluorescent in situ hybridisation (FISH) was used to determine the presence or absence of genes at Xp21, and therefore define whether a contiguous gene deletion was present. Analysis was performed using a *DAX1* (*NR0B1*) probe (BAC RP11-1129A5), a GK probe (RP11-229K14), and a dystrophin probe (NRM52), which shares homology with exon 52 in the dystrophin gene. Using a dosage test, the copy number of each of the 79 exons of the dystrophin gene was measured to identify the extent of the deletion in the dystrophin gene, using the P034 and P035 DMD multiplex ligation dependent probe amplification kit from MRC-Holland. Linkage analysis using marker DI671 situated in intron 67 of the dystrophin gene was performed to identify the origin of the deletion.

### Glycerol kinase activity

Epstein–Barr virus (EBV) transformed leucocytes were grown in RPMI 1640 with l-glutamine and 10% fetal calf serum, and washed with phosphate buffered saline (PBS). Glycerol kinase (GK) activity was measured in the cells after 2–3 min pre-treatment by digitonin using the GK catalysed conversion of [U-14C]glycerol to [U-14C]glycerol-3-phosphate as described before.^9^

Measurements were done in duplicates at two different protein concentrations from 0.1–1.2 g/l. Protein determination was performed on cell suspension dissolved in 0.5 mol/l NaOH according to the method of Lowry.^10^

### Methylation status of the androgen receptor

**Figure 1:**
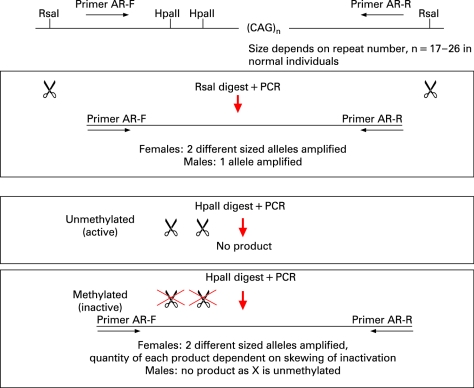
Method used to identify skewed X inactivation using two restriction enzymes (RsaI, which is methylation insensitive, and HpaII, which is methylation sensitive) on DNA amplified from the androgen receptor gene, which contains a variable number of CAG repeats and allow identification of parental DNA origin. AR, androgen receptor; PCR, polymerase chain reaction.

**Figure 2:**
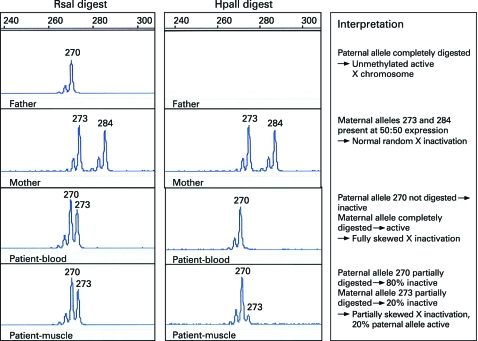
Genescanner traces for RsaI and HpaII digestions of paternal, maternal and proband DNA. A peak is produced for each androgen receptor (AR) allele amplified. The size of the allele is determined by the number of repeats within the AR gene. The area under the peak indicates the degree of amplification of that allele.

Two DNA aliquots from each family member were digested with restriction enzymes: one was HpaII, a methylation sensitive enzyme; and the other was the methylation insensitive enzyme, RsaI, which was used as a control for input DNA (fig 1). The digests were boiled for 10 min to inactivate the restriction enzymes and the products of digestion were used in polymerase chain reactions (PCRs) to selectively amplify a polymorphic CAG repeat within the androgen receptor (AR) gene. The two AR alleles, which differ in size in mother and father, were then visualised, sized and quantified using an ABI 3100 Genotyper (fig 2). The size of the allele indicates parental origin and the area under the peak indicates the degree of amplification of the allele.

HpaII digestion is blocked by methylation and therefore, in females, only the active (unmethylated) copies of the X chromosome are digested, and no PCR product is amplified. Methylated AR sequence will be amplified by HpaII. These digested copies do not amplify effectively, thus the PCR product generated from an HpaII digested sample represents the methylated (inactive) X chromosome.

The ratio of active (unmethylated) to inactive (methylated) AR alleles is a measure of the degree of X inactivation.^11^ This is said to be skewed when the ratio of peaks differs significantly from 50:50, generally by more than 70:30.

### Hormone assays

Renin activity was measured using DiaSorin radioimmunoassay (RIA) kit, (Saluggia, VC, Italy), and serum aldosterone values were determined using an in house RIA (Leeds General Infirmary, UK). ACTH and cortisol concentrations were measured using a Siemens immunoassay (Immunolite 2000, Llanberis, North Wales, UK).

## RESULTS

**Figure 3:**
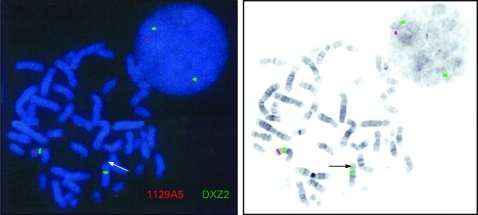
Fluorescent in situ hybridisation (FISH) analysis demonstrating deletion at Xp21.2. A signal was visible on only one X chromosome using probe 1129A5 (shown as red). Two signals from the centromeric X chromosome probe DXZ2 were visible (shown as green).

FISH analysis confirmed the patient had a gene deletion involving DAX-1 at Xp21.2, with a signal visible on only one X chromosome (fig 3). FISH testing carried out on maternal chromosomes showed the presence of two intact genes suggesting that either the child had a de novo deletion or there was parental gonadal mosaicism. FISH testing using a GK probe confirmed that a signal was present on only one chromosome, indicating that the GK gene was also deleted in the patient. The IL1RAPL1 locus downstream of DAX-1 was intact in this individual indicating that this was not the cause of her developmental delay.

Additional FISH studies using the dystrophin probe NRM52 demonstrated signals on both the patient’s X chromosomes, implying that any deletion in the dystrophin gene had occurred distal to exon 52.

Dosage analysis used to measure the copy number of each of the 79 dystrophin exons confirmed that the deletion involved exons 61 to 79, which would cause a DMD phenotype in males.

**Figure 4:**
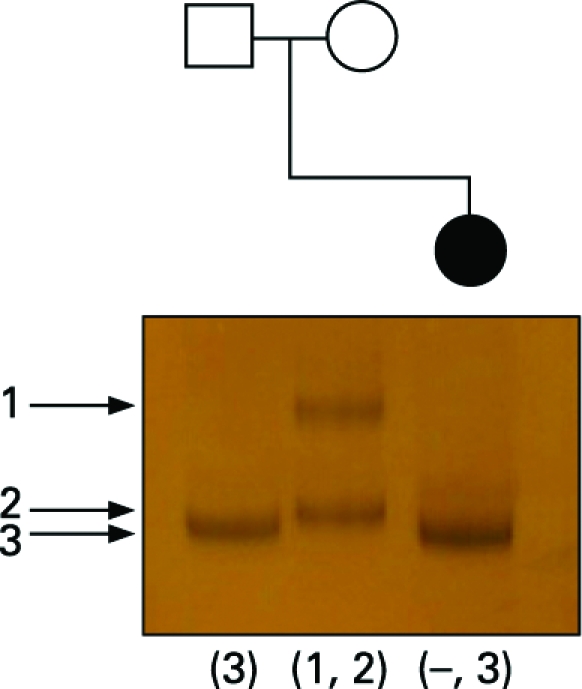
Linkage analysis in the parents and proband using marker DI671 situated in intron 67 of the dystrophin gene and therefore within the area deleted in the proband. Only one allele is detected in the proband and this is equivalent in size to her father’s marker at this locus, confirming the deletion is carried on her maternally inherited X chromosome.

Linkage analysis, using marker DI671 situated in intron 67 of the dystrophin gene and therefore within the region deleted in the patient, was performed. Only one allele was detected in the patient, corresponding to the paternal allele, confirming that the de novo deletion was carried on her maternally inherited X chromosome (fig 4).

### Glycerol kinase activity

The level of GK activity was assessed in the proband’s EBV transformed leucocytes compared to that in a normal control and found to be <1%, indicating complete inactivation of the gene on the normal paternal allele.

### X inactivation studies

Digestion with RsaI showed the proband had inherited a 270 bp AR allele from her father and 273 bp allele from her mother (fig 2). Digestion with HpaII showed that in the proband’s leucocyte DNA the paternal X chromosome was completely methylated (inactive), while the maternal chromosome, which carries the contiguous gene deletion, was completely unmethylated (active). In muscle there was a different pattern following HpaII digestion with both paternal and maternal methylated (inactive) alleles detected. The ratio of maternal to paternal allele expression was estimated at 80:20, indicating partially skewed X inactivation. Thus, in muscle approximately 20% of the paternal X chromosome was active.

## DISCUSSION

X-linked AHC is one of the most common causes of primary adrenal failure in boys. While most cases are due to point mutations in the *DAX1* (*NR0B1*) gene, approximately one third of cases are due to *DAX1* deletions, often as part of a contiguous gene deletion syndrome involving the dystrophin and GK genes. DMD is caused by a deficiency in dystrophin, a membrane associated protein found in muscle cells and neurones. This results in progressive muscle weakness in childhood, together with cardiomyopathy and respiratory problems. The signs of GKD are highly variable, ranging from no obvious phenotype with asymptomatic pseudohypertriglyceridaemia due to hyperglycerolaemia through to psychomotor retardation, growth delay, bone dysplasia, hypoglycaemia, ketoacidosis and seizures.^12^ ^13^ The signs of GKD in association with a contiguous gene deletion syndrome can be difficult to separate from the effects of the other conditions.

Thus routine screening for DMD (checking creatinine kinase concentrations) and GKD (checking urine concentrations of glycerol) should be carried out in any male presenting neonatally with AHC, as clinical signs of these associated disorders will not be apparent until later in infancy.

X-linked conditions, such as AHC, GKD and DMD, are usually found only in males since females can express sufficient levels of protein from the genes on their active X chromosome. If AHC is diagnosed in a female, it is important to establish whether there is a normal female karyotype, as females with 46XY karyotypes or Turner syndrome can manifest X-linked conditions.^14^^–^17 In the presence of a 46XX karyotype, autosomal recessive causes of AHC (such as StAR, SF1, (*NR5A1*) mutations) should be considered.^18^ ^19^

This case indicates the importance of considering X-linked AHC in female patients. We have confirmed that the cause of this girl’s adrenal insufficiency was due to a *DAX1* deletion on her maternal allele with skewed inactivation of her normal paternal allele. Once X-linked AHC has been confirmed in any patient, male or female, DMD and GKD should also be considered. The difference in disease severity between AHC and DMD in this child was due to the variability in the level of inactivation of the X chromosome in different tissues. The presence of functional dystrophin gene in muscle, albeit at 20% of normal levels, has resulted in a relatively mild clinical manifestation of DMD. At presentation, this child had notably abnormal electrolytes consistent with severe salt wasting, but a less severe deficiency of cortisol. Thus, mineralocorticoid insufficiency may be the predominant presenting feature of X-linked AHC, although glucocorticoid deficiency usually emerges with time.^20^ ^21^

Although most female carriers of X-linked diseases are asymptomatic, this is not always the case. Between 2.5–7.8% of female carriers of dystrophin gene mutations have been reported to have clinical symptoms.^22^ ^23^ The severity of symptoms in female carriers varies from muscle pain and cramp on exertion to severe muscle weakness leading to wheelchair dependency.^24^ Symptoms in carriers of DMD are usually thought to be due to skewed X inactivation,^25^ but they may also be due to Turner syndrome or X-autosomal translocations with a break point at Xp21 that disrupts the dystrophin gene and is associated with preferential inactivation of the non-translocated chromosome.^24^ Differences in X inactivation between tissues may also occur and result in dilated cardiomyopathy in females without skeletal muscle weakness.^26^ ^27^ However, the relationship between clinical phenotype, dystrophin expression and degree of X inactivation is not simple^28^; female patients can present with muscular dystrophy and normal X inactivation.^29^

In this case, X inactivation studies in DNA derived from peripheral blood mononuclear cells and from muscle tissue have demonstrated differences in the degree of skewing between the two tissues. There was no active paternally derived X chromosome in blood, and presumably the adrenal gland, giving rise to the neonatal presentation with salt losing adrenal failure. However, as there was some function of her normal paternal X chromosome in muscle, this patient had relatively mild signs of DMD.

Skewed X inactivation may be due to primary stochastic or genetic processes, or be secondary to cell selection during development.^30^ The initial choice of which X chromosome to inactivate is thought to be random, with subsequent skewing occurring as a consequence of post-inactivation selection against cells in which the active X chromosome is abnormal.^31^ This phenomenon is observed in X:autosome translocations^32^ and in carriers of a number of X-linked disorders, such as ATRX syndrome.^33^ Positive selection for cells carrying a mutated active X chromosome has also been reported.^34^

The X inactivation process is controlled by the X inactivation centre (Xic) on the proximal long arm of the X chromosome. Mutations in the XIST gene at Xq13.2 have been reported to cause familial non-random X chromosome inactivation. The XIST locus has not been analysed in our patient, but does not lie close to the deleted region. Other loci on the X chromosome linked to familial skewing of X inactivation have been reported, suggesting that these may be controlling the choice of X chromosome in the inactivation process or subsequent survival of cells during proliferation.^35^ ^36^

Skewed X inactivation occurring by chance appears to be rare, but has been reported in a series of phenotypically normal females. Skewing to the extent of >80:20 was observed in 8% of cases and to >95:5 in 0.8% of cases.^37^ There is no obvious explanation for the skewed X inactivation in our case; this is likely to represent a chance event as there is no apparent reason for cells containing the mutated maternal X chromosome to be positively selected during development. We did, however, find differences in X inactivation between tissues. This infers that the greater proportion of active paternal X chromosomes seen in muscle compared to leucocytes may be due to a selection bias against the maternal X chromosome.

### Conclusion

A *DAX1* mutation or deletion with skewed X inactivation should be considered in the differential diagnosis in 46XX females who present with neonatal adrenal insufficiency. Tests to identify dystrophin and GK gene deletions should also be undertaken to define whether a larger contiguous gene deletion is present. Variation in the level of X inactivation between tissues in girls with a contiguous gene deletion syndrome may account for differences in the severity of the disease manifestations.

## 

Obtained.

## References

[1] Seminara SB Achermann JC Genel M Jameson JL Crowley WF Jr X-linked adrenal hypoplasia congenita: a mutation in DAX1 expands the phenotypic spectrum in males and females. J Clin Endocrinol Metab 1999;84:4501–9 10599709 10.1210/jcem.84.12.6172

[2] Fujieda K Tajima T Molecular basis of adrenal insufficiency. Pediatr Res 2005;57 5 Pt 2:62R–9R 10.1203/01.PDR.0000159568.31749.4D 15817507

[3] Achermann JC The role of SF1/DAX1 in adrenal and reproductive function. Ann Endocrinol (Paris) 2005;66:233–9 15988384 10.1016/s0003-4266(05)81755-x

[4] Muscatelli F Strom TM Walker AP Zanaria E Recan D Meindl A Bardoni B Guioli S Zehetner G Rabl W Mutations in the DAX-1 gene give rise to both X-linked adrenal hypoplasia congenita and hypogonadotropic hypogonadism. Nature 1994;372:672–6 7990958 10.1038/372672a0

[5] Bartley JA Patil S Davenport S Goldstein D Pickens J Duchenne muscular dystrophy, glycerol kinase deficiency, and adrenal insufficiency associated with Xp21 interstitial deletion. J Pediatr 1986;108:189–92 3003318 10.1016/s0022-3476(86)80980-5

[6] Cole DE Clarke LA Riddell DC Samson KA Seltzer WK Salisbury S Congenital adrenal hypoplasia, Duchenne muscular dystrophy, and glycerol kinase deficiency: importance of laboratory investigations in delineating a contiguous gene deletion syndrome. Clin Chem 1994;40 11 Pt 1:2099–103 7955386

[7] Lin L Gu WX Ozisik G To WS Owen CJ Jameson JL Achermann JC Analysis of DAX1 (NR0B1) and steroidogenic factor-1 (NR5A1) in children and adults with primary adrenal failure: ten years’ experience. J Clin Endocrinol Metab 2006;91:3048–54 16684822 10.1210/jc.2006-0603 PMC1865080

[8] Zhang YH Huang BL Niakan KK McCabe LL McCabe ER Dipple KM IL1RAPL1 is associated with mental retardation in patients with complex glycerol kinase deficiency who have deletions extending telomeric of DAX1. Hum Mutat 2004;24:273 15300857 10.1002/humu.9269

[9] Sjarif DR Hellerud C van Amstel JK Kleijer WJ Sperl W Lacombe D Sass JO Beemer FA Duran M Poll-The BT Glycerol kinase deficiency: residual activity explained by reduced transcription and enzyme conformation. Eur J Hum Genet 2004;12:424–32 15026783 10.1038/sj.ejhg.5201172

[10] Lowry OH Rosebrough NJ, Farr Al, Randall RJ Protein measurement with the Folin phenol reagent. J Biol Chem 1951;193:265–75 14907713

[11] Allen RC Zoghbi HY Moseley AB Rosenblatt HM Belmont JW Methylation of HpaII and HhaI sites near the polymorphic CAG repeat in the human androgen-receptor gene correlates with X chromosome inactivation. Am J Hum Genet 1992;51:1229–39 1281384 PMC1682906

[12] Sargent CA Kidd A Moore S Dean J Besley GT Affara NA Five cases of isolated glycerol kinase deficiency, including two families: failure to find genotype:phenotype correlation. J Med Genet 2000;37:434–41 10851254 10.1136/jmg.37.6.434 PMC1734616

[13] Sjarif DR Sinke RJ Duran M Beemer FA Kleijer WJ Ploos van Amstel JK Poll-The BT Clinical heterogeneity and novel mutations in the glycerol kinase gene in three families with isolated glycerol kinase deficiency. J Med Genet 1998;35:650–6 9719371 10.1136/jmg.35.8.650 PMC1051390

[14] Katayama Y Tran VK Hoan NT Zhang Z Goji K Yagi M Takeshima Y Saiki K Nhan NT Matsuo M Co-occurrence of mutations in both dystrophin- and androgen-receptor genes is a novel cause of female Duchenne muscular dystrophy. Hum Genet 2006;119:516–9 16528518 10.1007/s00439-006-0159-4

[15] Kelsey G Monagle P Barnes C Delayed diagnosis of congenital factor IX deficiency (Christmas disease) in a girl with Turner’s syndrome. Clin Lab Haematol 2006;28:355–6 16999730 10.1111/j.1365-2257.2006.00810.x

[16] Massa G Vanderschueren-Lodeweyckx M Spondyloepiphyseal dysplasia tarda in Turner syndrome. Acta Paediatr Scand 1989;78:971–4 2603728 10.1111/j.1651-2227.1989.tb11189.x

[17] Wulfsberg EA Skoglund RR Duchenne muscular dystrophy in a 46 XY female. Clin Pediatr (Phila) 1986;25:276–8 3698449 10.1177/000992288602500509

[18] Biason-Lauber A Schoenle EJ Apparently normal ovarian differentiation in a prepubertal girl with transcriptionally inactive steroidogenic factor 1 (NR5A1/SF-1) and adrenocortical insufficiency. Am J Hum Genet 2000;67:1563–8 11038323 10.1086/316893 PMC1287931

[19] Bose HS Sugawara T Strauss JF III Miller WL The pathophysiology and genetics of congenital lipoid adrenal hyperplasia. International Congenital Lipoid Adrenal Hyperplasia Consortium. N Engl J Med 1996;335:1870–8 8948562 10.1056/NEJM199612193352503

[20] Verrijn Stuart AA Ozisik G de Vroede MA Giltay JC Sinke RJ Peterson TJ Harris RM Weiss J Jameson JL An amino-terminal DAX1 (NROB1) missense mutation associated with isolated mineralocorticoid deficiency. J Clin Endocrinol Metab 2007;92:755–61 17164309 10.1210/jc.2005-2429

[21] Wiltshire E Couper J Rodda C Jameson JL Achermann JC Variable presentation of X-linked adrenal hypoplasia congenita. J Pediatr Endocrinol Metab 2001;14:1093–6 11592565 10.1515/jpem-2001-0804

[22] Norman AM Upadhyaya M Thomas NS Roberts K Harper PS Duchenne muscular dystrophy in Wales: impact of DNA linkage analysis and cDNA deletion screening. J Med Genet 1989;26:565–71 2810340 10.1136/jmg.26.9.565 PMC1015695

[23] Penn AS Lisak RP Rowland LP Muscular dystrophy in young girls. Neurology 1970;20:147–59 5460703 10.1212/wnl.20.2.147

[24] Hoogerwaard EM Bakker E Ippel PF Oosterwijk JC Majoor-Krakauer DF Leschot NJ van Essen AJ Brunner HG van der Wouw PA Wilde AA de Visser M Signs and symptoms of Duchenne muscular dystrophy and Becker muscular dystrophy among carriers in the Netherlands: a cohort study. Lancet 1999;353:2116–9 10382696 10.1016/s0140-6736(98)10028-4

[25] Richards CS Watkins SC Hoffman EP Schneider NR Milsark IW Katz KS Cook JD Kunkel LM Cortada JM Skewed X inactivation in a female MZ twin results in Duchenne muscular dystrophy. Am J Hum Genet 1990;46:672–81 2180286 PMC1683658

[26] Hoogerwaard EM van der Wouw PA Wilde AA Bakker E Ippel PF Oosterwijk JC Majoor-Krakauer DF van Essen AJ Leschot NJ de Visser M Cardiac involvement in carriers of Duchenne and Becker muscular dystrophy. Neuromuscul Disord 1999;9:347–51 10407858 10.1016/s0960-8966(99)00018-8

[27] Mirabella M Servidei S Manfredi G Ricci E Frustaci A Bertini E Rana M Tonali P Cardiomyopathy may be the only clinical manifestation in female carriers of Duchenne muscular dystrophy. Neurology 1993;43:2342–5 8232953 10.1212/wnl.43.11.2342

[28] Matthews PM Benjamin D Van BI Squier MV Nicholson LV Sewry C Barnes PR Hopkin J Brown R Hilton-Jones D Muscle X-inactivation patterns and dystrophin expression in Duchenne muscular dystrophy carriers. Neuromuscul Disord 1995;5:209–20 7633186 10.1016/0960-8966(94)00057-g

[29] Bushby KM Goodship JA Nicholson LV Johnson MA Haggerty ID Gardner-Medwin D Variability in clinical, genetic and protein abnormalities in manifesting carriers of Duchenne and Becker muscular dystrophy. Neuromuscul Disord 1993;3:57–64 8329890 10.1016/0960-8966(93)90042-i

[30] Brown CJ Robinson WP The causes and consequences of random and non-random X chromosome inactivation in humans. Clin Genet 2000;58:353–63 11140834 10.1034/j.1399-0004.2000.580504.x

[31] Migeon BR Non-random X chromosome inactivation in mammalian cells. Cytogenet Cell Genet 1998;80:142–8 9678349 10.1159/000014971

[32] Schmidt M Du SD Functional disomies of the X chromosome influence the cell selection and hence the X inactivation pattern in females with balanced X-autosome translocations: a review of 122 cases. Am J Med Genet 1992;42:161–9 1733164 10.1002/ajmg.1320420205

[33] Muers MR Sharpe JA Garrick D Sloane-Stanley J Nolan PM Hacker T Wood WG Higgs DR Gibbons RJ Defining the cause of skewed X-chromosome inactivation in X-linked mental retardation by use of a mouse model. Am J Hum Genet 2007;80:1138–49 17503331 10.1086/518369 PMC1867101

[34] Migeon BR Moser HW Moser AB Axelman J Sillence D Norum RA Adrenoleukodystrophy: evidence for X linkage, inactivation, and selection favoring the mutant allele in heterozygous cells. Proc Natl Acad Sci USA 1981;78:5066–70 6795626 10.1073/pnas.78.8.5066 PMC320333

[35] Pegoraro E Whitaker J Mowery-Rushton P Surti U Lanasa M Hoffman EP Familial skewed X inactivation: a molecular trait associated with high spontaneous-abortion rate maps to Xq28. Am J Hum Genet 1997;61:160–70 9245997 10.1086/513901 PMC1715880

[36] Cau M Addis M Congiu R Meloni C Cao A Santaniello S Loi M Emma F Zuffardi O Ciccone R Sole G Melis MA A locus for familial skewed X chromosome inactivation maps to chromosome Xq25 in a family with a female manifesting Lowe syndrome. J Hum Genet 2006;51:1030–6 16955230 10.1007/s10038-006-0049-6

[37] Amos-Landgraf JM Cottle A Plenge RM Friez M Schwartz CE Longshore J Willard HF X chromosome-inactivation patterns of 1,005 phenotypically unaffected females. Am J Hum Genet 2006;79:493–9 16909387 10.1086/507565 PMC1559535

